# Competitive Sorption and Carrier-Facilitated Transport of Organic Polymers by Clay Minerals in Limestone Media: Experimental Evidence and Numerical Analysis

**DOI:** 10.1021/acs.est.5c09934

**Published:** 2026-01-28

**Authors:** Nimo Kwarkye, Thomas Ritschel, Andreas Pihan, Kai U. Totsche

**Affiliations:** 1 Department of Hydrogeology, 9378 Friedrich-Schiller University Jena, Burgweg 11, 07749 Jena, Germany; 2 Cluster of Excellence Balance of the Microverse, Friedrich Schiller University Jena, Fürstengraben 1, 07743 Jena, Germany

**Keywords:** mobile sorbents, carrier-facilitated transport, inverse modeling, polymer tracers, montmorillonite, carrier-assisted transport

## Abstract

Clay minerals acting as carriers in permeable media can facilitate the transport of less mobile pollutants, including radionuclides, heavy metals, pesticides, polycyclic aromatic hydrocarbons, and organic polymers. In a colloidal dispersed state, clays can increase the mean transport velocity of adsorbed pollutants by several orders of magnitude. Hence, delineated groundwater protection zones and riverbank filtration systems based on pollutant mobility may fail if carrier-facilitated transport is neglected. Yet, leveraging carrier-assisted transport for the controlled release of pollutants from soil may open new remediation opportunities. However, the determination and parametrization of competitive adsorption on mobile and immobile sorbents, crucial for carrier-facilitated transport, are often obscured by the interplay among numerous interaction processes in natural porous media. We present experiments in which montmorillonite minerals facilitated the transport of polyethylene glycol (PEG), resulting in a 10-fold increase in mean transport velocity in limestone media. PEG’s high affinity for montmorillonite enabled the mobilization of PEG already adsorbed at immobile interfaces. Additionally, model simulations suggested that high flow rates, for example, due to ponding infiltration, were favorable for carrier-facilitated transport, even for weak adsorption to mobile sorbents. We developed a sequential experimental protocol that permits identification of crucial factors controlling competitive sorption during carrier-facilitated transport.

## Graphical abstract

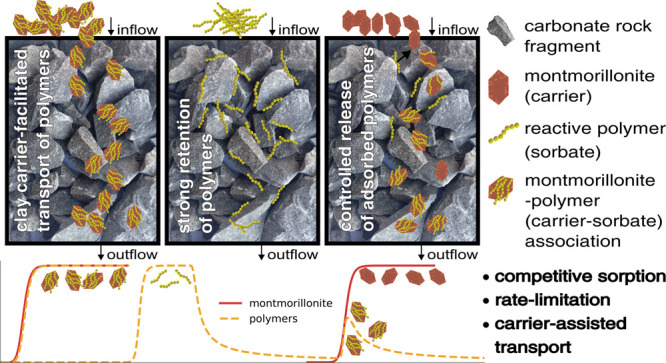

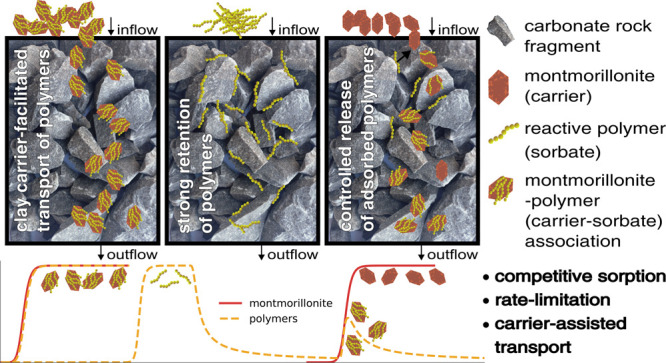

## 1 Introduction

Soil’s mobile phase may contain a significant proportion of clay minerals,^1,2^ often released in situ during weathering and pedogenesis^3,4^ or due to changes in physicochemical conditions, such as ionic strength.^5^ With their high surface area and reactivity, clay minerals can adsorb a wide variety of organic and inorganic substances in large quantities. In a colloidally dispersed state, mobile clays and, hence, the components adsorbed to their surface are often as mobile as the fluid, sometimes even with a higher average velocity due to pore size exclusion effects.^6^ Consequently, mobile clay minerals may govern the mobility of a wide range of substances that are otherwise considered immobile in aqueous solutions.^7^ This carrier-facilitated transport mechanism has been documented for harmful pollutants such as arsenic,^8^ trace elements,^9^ and heavy metals.^10^ However, there is growing interest in also exploring carrier-facilitated transport for targeted delivery and remediation, e.g., the use of organic polymers to facilitate the controlled release of agrochemicals,^11^ delivery of polymer-associated pesticides,^12^ and polymer-mediated carbon storage.^13^ The effectiveness of such technologies demands an application targeted to the specific reactions that control carrier-facilitated transport. Without a robust quantification of transport parameters that, e.g., control competitive sorption between mobile and immobile sorbents in soil, and a model accounting for interactions with mobile carriers, such an assessment is likely to fail. Yet, transport models based on the advection-dispersion equation (ADE)^14,15^ that employ complex multisite adsorption models usually only consider single-solute transport and neglect the potential effects of colloidal or carrier-facilitated transport. In contrast, models of colloid transport often assume equilibrium colloid deposition processes^16^ but nonequilibrium deposition reactions are common in natural systems.^17,18^

Given this, Knabner et al.^19^ introduced a transport model for predicting carrier-facilitated transport of hydrophobic organic carbon on dissolved organic matter that accounted for the intricate dynamics of sorbate sorption to mobile and immobile sorbents, including (non)­equilibrium sorption and deposition processes.^20,21^ This represented competition between mobile and immobile sorbents for sorbate adsorption.^22,23^ However, the multitude of mechanisms involved in nonlinear and nonequilibrium sorption^17,18^ results in a large number of model parameters, even for single-solute transport, which more than doubles when carrier-facilitated transport is also considered. Consequently, equifinality between transport processes produces nonunique parameter estimates with significant uncertainties when determined with inverse modeling.^24,25^ In these situations, independent model parametrization^26^ and an adapted experimental protocol are required to reduce parameter uncertainty and eliminate parameter correlations by eliciting an experimental response that is uniquely sensitive to a specific, noncorrelated set of parameters.^27^ Yet, experimental data for such an assessment can be obtained only using a tracer alien to the porous medium and tailored to a particular sorbent, which shows the most informative interaction.

Recently, tailor-made poly­(ethylene glycol) (PEG) has been employed as a reactive tracer with a strong affinity for clay minerals such as Illite, montmorillonite, and kaolinite that constitute a potentially mobile fraction in soil aqueous suspensions.^26,28^ The affinity of PEG for clay minerals has been explored as an interfacial tracer to identify and quantify reactive and immobile clay minerals in limestone media,^29^ rendering tailored PEG a promising tracer also for identifying and quantifying mobile clay colloids. Furthermore, other PEG derivatives are employed in remediation technologies, and understanding PEG’s transport behavior in the presence of a mobile sorbent is crucial. As such, the use of PEG could yield insights into carrier-facilitated transport regarding both the carrier and the sorbate in the subsurface. Therefore, we used fluorescently labeled PEG as a tracer in column experiments to explore two aspects of carrier-facilitated transport. First, we considered the change in PEG transport behavior when introduced with mobile montmorillonite minerals to determine whether interaction with mobile clays limits a sorbate’s potential for retention at immobile surfaces. Second, we illustrate the potential of mobile clays to compete with sorbates already attached to porous media surfaces by introducing montmorillonite into porous media already saturated with PEG. In each experiment, a unique response was elicited for a set of transport parameters, enabling the successive parametrization of a carrier-facilitated transport model that reduced model uncertainty and enhanced the robustness of predictions. As a substrate, we used limestone collected from the Hainich Critical Zone Exploratory that show a considerable fraction of available immobile sorbents.^3^ Furthermore, carbonates serve as host rock for large aquifer units^30,31^ and make up a substantial percentage of the ice-free land on the Earth.^32^ Their capacity to hold large quantities of water and provision of habitats for numerous subsurface organisms^33^ make carbonate media essential to the earth’s critical zone. However, their vulnerability to pollution demands an extensive assessment of fluid flow and the transport behavior of dissolved and suspended compounds.

## 2 Materials and Methods

### 2.1 Mobile and Immobile Sorbents

We used fresh, unweathered limestone material obtained from the stratigraphic unit of the Hainich carbonate aquifer in all column experiments. A detailed description of the applied limestone material can be found in Kwarkye et al.^29^ and Ritschel et al.^3^ In brief, limestone materials are predominantly composed of calcite and dolomite but also contain about 5 wt % of clay minerals embedded in the rock matrix and are exposed during limestone weathering. Limestone material was crushed to 2 mm-5 mm clasts and thoroughly rinsed with ultrapure water (Milli-Q, Integral 5, Elix Technology Inside, Merck Millipore, Darmstadt, Germany) to remove any remnants resulting from the crushing process.

Montmorillonite extracted and purified from bentonite (MX80) was used as a mobile sorbent in column experiments and substrate in batch experiments. Details of the montmorillonite extraction protocol are provided in the Supporting Information. Once extracted, montmorillonite was treated with sodium chloride (NaCl, Roth, Germany) to exchange calcium ions for sodium ions, yielding sodium montmorillonite. The average size of montmorillonite was determined using dynamic light scattering (DLS) (Nano ZS, Malvern Panalytical Ltd., Malvern, UK). Additionally, scanning electron microscopy (SEM-EDX ULTRA PLUS, Zeiss AG, Germany) measurements were conducted to characterize physical features like the shape and structure of montmorillonite.

### 2.2 Transport and Adsorption Experiments

Batch experiments were conducted in triplicate to explore the adsorption characteristics of polyethylene glycol (PEG) to limestone media and montmorillonite colloids. A detailed description of the PEG synthesis can be found in Kwarkye et al.^29^ Tubes (50 mL, VWR, Radnor Corporate Center, USA) were filled with 2 mg of montmorillonite and mixed with 25 mL of a PEG solution (in 50 mg L^–1^, 40 mg L^–1^, 30 mg L^–1^, 20 mg L^–1^, 10 mg L^–1^, and 5 mg L^–1^). Unless indicated otherwise, degassed ultrapure water was used as the background medium for all treatments and tracer solutions to prevent colloidal aggregation resulting from increased ionic strength. Subsequently, the samples were shaken at 101 rpm for 72 h at 20 °C. A set of batches without substrate was processed identically for each substrate as blanks.

A series of column experiments was conducted to investigate carrier-facilitated transport. Columns were set up in duplicates, referred to as column I and column II, with the same treatment (replicates) in all column experiments as follows. The columns (borosilicate glass, inner diameter: 3.7 cm, length: 7.7 cm) were dry-packed with limestone, applying a 1 cm stepwise compaction to achieve a homogeneous distribution with bulk densities ranging from 1.60 to 1.73 g cm^–3^. Bulk density, water content, and applied flow rate were determined gravimetrically (Supporting Information Table 1). The columns were saturated from bottom to top at a rate of 0.5 pore volumes (pv) per day to determine the volumetric water content. Conservative transport was assessed with 10 mM NaCl for three pv at a rate of approximately two pv per day. The columns were then rinsed again with ultrapure water until the background electrical conductivity was reached. An individual set of columns was then used for each of the following scenarios:

Scenario I: columns were fed with 50 mg L^–1^ PEG solution. This scenario represents the interactions of a mobile sorbate in natural porous media where sorbents are mostly immobile. It was used to reveal PEG’s transport behavior in the absence of carrier-facilitated transport.

Scenario II: columns were fed with 1 g L^–1^ montmorillonite suspension. This scenario illustrates the interactions of a mobile sorbent in the same porous media.

Scenario III: columns were fed with a suspension containing 1 g L^–1^ montmorillonite and 50 mg L^–1^ PEG, which instantaneously adsorbed to montmorillonite upon mixing due to the strong affinity of PEG toward montmorillonite. The ratio of PEG concentration to montmorillonite concentration of 5:100 was chosen to match the ratio of organic compounds to mobile particles often found in soil seepage.^1^ The high content of montmorillonite resulted in all PEG being adsorbed before the suspension was introduced into the limestone media as seen in the absence of PEG after filtration. This scenario illustrates how mobile sorbents influence sorbate transport, particularly when they exhibit a high affinity for the sorbate.

Scenario IV: columns were presaturated with PEG by percolating with 50 mg L^–1^ PEG until a plateau was reached in the effluent concentration. Two flow interrupts were conducted at high PEG concentration gradients to allow kinetically limited PEG interactions to be equilibrated. This was followed by rinsing back to the background level in the effluent, while observing flow interruptions to account for nonequilibrium desorption. Considering the strong desorption hysteresis of PEG at limestone media with exposed clay minerals,^29^ a significant fraction of PEG is expected to be retained in the columns even if the column effluents are void of PEG. This was validated by performing a second breakthrough while observing flow interruptions, after which the columns were rinsed again with the background solution. Afterward, the columns were fed with a 1 g L^–1^ montmorillonite suspension to demonstrate its ability to remobilize and transport PEG already associated with surfaces. Details of the experimental protocol, pulse durations, and flow interruptions are presented in Table 2 of the Supporting Information.

### 2.3 Data Acquisition and Analysis

We exploited the initiation site of PEG for its quantification by UV–vis spectroscopy (Varian Cary 50, Agilent Technologies Inc., Santa Clara, CA, USA). Samples were excited from 240 to 370 nm at 1 nm resolution, corresponding to the excitation wavelength of the fluorene moiety used as an initiation site during PEG synthesis. As shown by Kwarkye et al.^29^ for PEG fluorescence, this permits a linear calibration of UV-vis signals within the applied concentration range. A negligible number of particles was released in scenario I, where montmorillonite was absent, such that the observed turbidity spectra in scenarios II, III, and IV represented a unique signature of montmorillonite in our experiments. This permitted a linear UV-vis signal calibration within the applied montmorillonite concentration range. In the column experiments, samples were measured at 5 min intervals by connecting the column outflow to flow-through cells in the UV-vis spectrophotometer. The data were decomposed into spectra associated with PEG and montmorillonite using non-negativity-constrained matrix factorization.^34^ Both spectral components were calibrated to signals of pure PEG solutions (50, 30, 20, 10, 2, 1, and 0.5 mg L^–1^) and pure montmorillonite (1000, 500, 200, 100, and 50 mg L^–1^) using a linear function.

### 2.4 Predicting Carrier-Facilitated Transport

A detailed derivation of the carrier-facilitated transport model is presented in Totsche,^21^ Knabner et al.,^19^ and Totsche et al.^20^ In brief, the transport of PEG either bound to montmorillonite or freely mobile in solution was modeled using an advection-dispersion equation with multisite adsorption for both equilibrium and nonequilibrium sites as follows

$$\begin{array}{c} \frac{\theta \partial c_{T}}{\partial t}+\frac{\rho_{T}\partial s_{T}^{eq}}{\partial t}=\frac{\theta D\partial^{2}c_{T}}{\partial x^{2}}-\frac{q\partial c_{T}}{\partial x}-\\ \left(1-\beta \right)\alpha_{f}\rho \left[s_{f}^{neq}-\gamma_{f}\right]-\alpha_{b}\rho \left[s_{b}^{neq}-\gamma_{b}\right]\\ c_{T}=c_{f}+c_{b} \end{array}$$ 1

where *c*_*T*_ is the concentration of PEG either carrier-associated or free in solution, $\frac{\partial }{\partial t}$ and $\frac{\partial }{\partial x}$ are temporal and spatial derivatives respectively, θ is the water content, ρ represents the bulk density of the limestone medium, *q* is the Darcy velocity, *D* is the apparent dispersion coefficient calculated as *D* = λ*q*/θ, using the dispersion length λ. Furthermore, α is a pseudo-first-order mass exchange coefficient, and *s* represents an adsorption isotherm. The bulk density of the porous medium was partitioned into fractions providing equilibrium and nonequilibrium adsorption sites for the adsorption of free PEG according to the adsorption coefficient β. Furthermore, γ is the PEG concentration at nonequilibrium sites. Subscripts *f* (free) and *b* (bound) indicate free and carrier-associated PEG parameters, respectively. Superscripts *eq* and *neq* also indicate adsorption isotherm parameters representing the sorption process at equilibrium and nonequilibrium sorption sites, respectively. The isotherm *s* for the adsorption of free and bound PEG to immobile surfaces were defined respectively as follows:

$$s_{f}=\frac{K_{f}c_{f}s_{\max}}{1+K_{f}c_{f}},s_{b}=K_{b}c_{b}$$ 2

where *s*_*f*_ and *s*_*b*_ (M M^–1^) are the adsorbed masses of free and carrier-associated PEG on immobile surfaces in equilibrium, *c*_*f*_ and *c*_*b*_ (M L^–3^) are the concentrations of free and bound PEG in solution, *K*_*f*_ (L^3^ M^–1^) is the Langmuir isotherm constant, *K*_*b*_ (L^3^ M^–1^) is a linear isotherm constant, and *s*_*max*_ (M M^–1^) is the PEG adsorption capacity of the immobile phase.

PEG bound to montmorillonite (*c*_*b*_) can be written in terms of free PEG (*c*_*f*_) by assuming PEG-montmorillonite associations contain a fixed mass of PEG and montmorillonite.

$$c_{b}=\pi \left(c_{f}\right)c_{m}$$ 3

where *c*_*m*_ is the concentration of montmorillonite in solution and π­(*c*_*f*_) is the adsorption isotherm representing PEG adsorption to montmorillonite.^19^ The interaction between PEG and its carrier montmorillonite was modeled using another Langmuir isotherm with *K* ^*mt*^ and *s*_*max*_^*mt*^ for the isotherm constant and PEG adsorption capacity of montmorillonite, respectively. Equations 3 and 1 constitute the final model representing PEG transport in the presence of montmorillonite.

However, these equations depend on montmorillonite concentration in solution, which was modeled with another advection-dispersion equation considering a colloid deposition process according to Knabner et al.^19^ as follows:

$$\frac{\theta \partial c_{m}}{\partial t}=\frac{\theta D\partial^{2}c_{m}}{\partial x^{2}}-\frac{q\partial c_{m}}{\partial x}-k\left(\Gamma c_{m}-\varphi \right)$$ 4

where *k* is the colloid deposition rate constant, Γ is the deposition frequency, and φ is the actual deposition, which was formulated as

$$\frac{\partial \varphi }{\partial t}=k\left(\Gamma c_{m}-\varphi \right)$$ 5

In summary, an assessment of PEG transport facilitated by montmorillonite requires specification of all transport parameters outlined in the equations above, i.e., parameters for PEG (non)­equilibrium adsorption to immobile clay minerals, adsorption parameters for adsorption to montmorillonite, and montmorillonite deposition parameters in the porous medium. This resulted in many parameters that could not be fitted simultaneously without encountering severe parameter uncertainties, correlations, and equifinality,^35^ compromising the approach. Therefore, the series of experimental scenarios described above was used to sequentially determine only a small fraction of uncorrelated parameters, individually from breakthrough data indicative of each parameter. Values of S_*max*_ were derived independently from batch experiments, as they are strongly correlated with the Langmuir adsorption coefficient K. The latter was derived from experimental breakthrough curves, as the values obtained in batch experiments cannot be readily transferred to data from experiments under flow and transport conditions.^36^ This approach, outlined below, permitted a robust prediction of transport in scenario IV.

### 2.5 Sequential Fitting Approach

First, PEG transport parameters describing transport in limestone media, independent of potential carriers, were determined inversely using data from scenario I. This reduced the fitted parameters to isotherm parameters accounting for PEG adsorption to the immobile phase. The Langmuir isotherm for the limestone’s PEG adsorption capacity parameter, *s*_*max*_, was obtained from the study by Kwarkye et al.,^29^ who used the identical sorbents and sorbates. The dispersion length was determined by fitting a conservative ADE transport model to NaCl breakthrough data collected in the columns prior to the actual PEG transport experiment (see Supporting Information Table 1 and Figure 3). In sum, the fitted parameters (Table 1) for PEG transport in scenario I reduced to the Langmuir isotherm constants representing the sorption process for equilibrium and nonequilibrium adsorption sites *K* ^*eq*^ and *K* ^*neq*^ respectively, the fraction of equilibrium adsorption sites β, and the pseudo first-order mass exchange coefficient α_*f*_.

**Table 1:** Results of Fitted Transport Parameters in Transport Scenarios^a^

Parameters	column I	column II
Scenario I		
*K* ^*eq*^ (L mg^–1^)	0.007 ± 0.0003	0.007 ± 0.0003
*K* ^*neq*^ (L mg^–1^)	2.57 ± 0.12	4.59 ± 0.18
β_*f*_ (−)	0.09 ± 0.002	0.15 ± 0.002
α_*f*_ (h^–1^)	0.007 ± 1.6 × 10^–5^	0.008 ± 2.0 × 10^–5^
Scenario II		
*k* (h^–1^)	0.03 ± 0.002	0.03 ± 0.002
Γ (L g^–1^)	0.0002 ± 8.0 × 10^–6^	0.0002 ± 9.0 × 10^–6^
Scenario III		
*K*_*b*_^*neq*^ (L g^–1^)	1.1 × 10^–4^ ± 4.6 × 10^–6^	9.8 × 10^–5^ ± 4.0 × 10^–6^
α_*b*_ (h^–1^)	0.089 ± 0.008	0.084 ± 0.007
*K* ^*mt*^ (L mg^–1^)	4.3 ± 0.17	14.9 ± 0.82

a Uncertainty of parameters is presented as 95% confidence interval.

Next, the montmorillonite transport parameters in the limestone medium were determined inversely from the turbidity data for scenario II. Specifically, the deposition frequency Γ and deposition rate constant k were fitted. The parameters from scenarios I and II were then used in scenario III to characterize the behavior of pure PEG and montmorillonite. As a result, only the parameters related to the association between PEG and montmorillonite remained to be determined. As scenario III provided breakthrough data for the suspended fraction via the turbidity observed in UV-vis spectra as well as the specific PEG signal, we derived the linear isotherm constant *K*_*b*_^*neq*^ from eq 2 and the pseudo first-order mass exchange coefficient α_*b*_ that describes the transport of the PEG-montmorillonite association from the turbidity data and the isotherm constant *K* ^*mt*^ for PEG adsorption on montmorillonite from the PEG breakthrough. This stepwise fitting approach minimizes ambiguities in multiparameter optimization by determining only a few parameters at a time. Finally, the obtained transport model parametrization was applied to predict cotransport in scenario IV without fitting.

Dimensionless parameters, including the Damköhler (Da) number and the retardation coefficient (R),^24^ were estimated to assess and compare transport in different scenarios. Additionally, mass balance equations were used to calculate tracer retention after they passed through the columns.^29^

### 2.6 Sensitivity Analysis

The transport model obtained in scenario III was applied in a sensitivity analysis to obtain sorption parameters crucial for competitive adsorption between montmorillonite and immobile clay minerals. For this, a set of forward simulations was generated for values K*=KmtKfneq of 0.001, 0.01, 0.1, 1, 10, 100, 1000, which represented the situation where the isotherm constants of mobile sorbents were 1000x less to 1000x greater than the isotherm constants of immobile sorbents. For each K* value, forward simulations were performed with smax*=smaxmtsmaxneq values given as 0.001, 0.01, 1, 10, 100, and 1000, also representing the condition where adsorption capacity of mobile sorbent is 1000x lower than adsorption capacity of immobile sorbents and vice versa. These sets of simulations were generated at Damköhler numbers (Da) 0.1, 1, 10, and 100, covering conditions where mass exchange reactions are 10 times slower to 100 times faster than the mean residence time.

## 3 Results and Discussion

### 3.1 Characterization of Montmorillonite and Comparison to Clay Minerals in Limestone Media

Montmorillonite particles depicted plate-like features and aggregated structures (see Supporting Information Figure 1). An average montmorillonite size of 440 ± 6 nm was measured using DLS, which decreased slightly to 430 ± 4 nm after transport through the columns, most likely due to colloid deposition and straining processes typically observed for colloidal transport.^16,37^ The FT-IR spectra of montmorillonite exhibited pronounced peaks at 3490 cm^–1^ and 1030 cm^–1^, corresponding to the OH and Si–O stretching, respectively (see Supporting Information Figure 1). The OH bands are attributed to surface hydroxyl groups at the edges of clay minerals. Additionally, siloxane surface groups in clay minerals manifest as Si–O bands in measured FT-IR spectra. As part of the smectite phyllosilicate group, Montmorillonite is a layered sheet clay mineral composed of silicon, aluminum, oxygen, hydrogen, and calcium or sodium.^38^

### 3.2 Decomposition of UV-Visible Spectra

PEG and montmorillonite concentrations in the aqueous phase were quantified from measured UV–vis spectra by a linear combination fit (LCF).^39,40^ PEG spectra were measured and used as one of the components in the linear combination fit. Montmorillonite quantification was based on the turbidity spectra of the effluent solution,^41^ which is characteristic of the average colloid size in the solution. In effect, processes such as colloid deposition and straining^16,37^ decrease the average particle size, lower solution turbidity, and reduce light-scattering intensity. The change in scattering intensity with particle size also modified the shape of the turbidity spectra. Consequently, when measured directly, the slightly larger montmorillonite particles produced a spectrum distinct from that obtained after transport through limestone media. To circumvent an improper representation of montmorillonite in the UV-vis spectra, montmorillonite was represented by two components, where one was measured in a stock solution and the other was obtained from UV–vis spectra of montmorillonite after passing the columns in scenario 2, where PEG was absent. Both components were then combined linearly to represent the total montmorillonite content. PEG and montmorillonite components (see Supporting Information Figure 2) were then applied in a linear combination fit to quantify PEG and montmorillonite in scenarios 1, 2, 3, and 4 with an explained variance of 99.7%.

### 3.3 PEG Adsorption on Clay Minerals

A strong adsorption of PEG to montmorillonite was observed in batch experiments (Figure 1) with an adsorption capacity of 303 ± 93 mg g^–1^. This adsorption capacity was more than 3 orders of magnitude higher than that of PEG adsorption reported in limestone media at 0.20–0.24 mg g^–1^ (Figure 1). The low adsorption capacity of limestone has been attributed to the limited exposure of reactive clay mineral surfaces in previous research.^29^ The applied PEG was nonionic and therefore only adsorbs to montmorillonite by forming hydrogen bonds via functional groups with siloxane surface groups^42^ or engage in acid–base interactions with surface silanol groups via the PEG ether group.^43^ Since these surface functional groups were also present in clay minerals found in limestone media, a similar PEG adsorption mechanism persisted in limestone media.^29^ However, the swelling property of montmorillonite, once solvated, may facilitate interlayer PEG adsorption, thereby further increasing the adsorption capacity for PEG.^44,45^ The adsorption in montmorillonite media was characterized by a sharp increase with an estimated isotherm constant of 12.4 ± 6.3 L mg^–1^, indicating a strong adsorption affinity to montmorillonite. The adsorption plateau occurred at an equilibrium concentration of 5 mg L^–1^ of PEG, beyond which there was no significant change in adsorption (Figure 1), which was reconstructed using the Langmuir adsorption isotherm with high accuracy.

**Figure 1:**
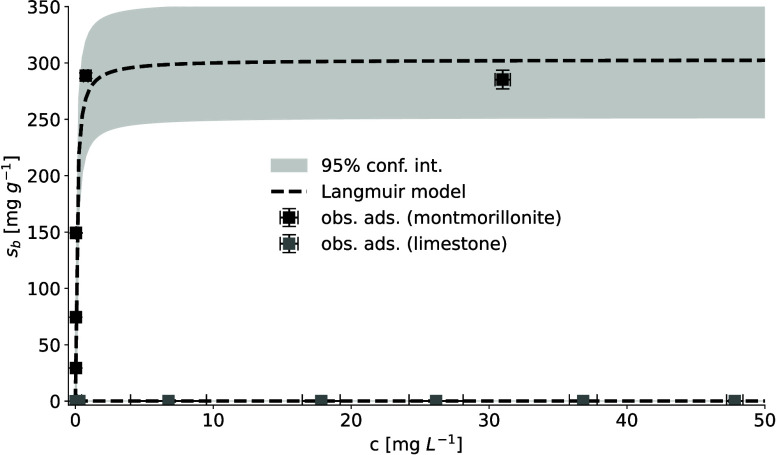
Measured PEG adsorption to montmorillonite suspension compared with PEG adsorption to limestone.^29^ Error bars represent the standard deviation of three experimental replicates. The reconstructed Langmuir adsorption isotherm is shown as a black dashed line. The uncertainty of the adsorption isotherm is given as a 95% confidence interval.

### 3.4 PEG Transport in Limestone Media (Scenarios I and IV)

We now discuss the transport of PEG in limestone media, as observed in scenarios I and IV, without the presence of mobile clays. In both scenarios, the first arrival of PEG at the column effluent was about 5 times later than that of NaCl, which behaved conservatively with a breakthrough around one exchanged pore volume (see Figure 2, Figure 3a, and Supporting Information Figure 3, respectively). While the center-of-mass of NaCl occurred at approximately one pore volume, that of PEG passed after more than 14 pore volumes of tracer were exchanged. Additionally, the shape of the first arrival wave of PEG appeared much more dispersed in scenario I (Figure 3a) than that of NaCl (see Supporting Information Figure 3). These observations indicate strong interactions between PEG and reactive surfaces in the limestone medium, as previously reported by Kwarkye et al.,^29^ who accounted for PEG adsorption to exposed immobile clay minerals in limestone media. Additionally, PEG’s response to immobile clay mineral exposition following limestone dissolution contributed to rate-limited adsorption, which was suspected to have resulted in the dispersed shape of the first arrival wave of PEG. In effect, the flow interruptions in scenario IV resulted in a sudden drop in PEG concentration at the effluent after flow resumed (Figure 2), indicating a strong rate-limited adsorption process.^18,46^ From the estimated mass balance, about 44–50.8 mg of PEG was applied to the columns, out of which about 19.8–20.4 mg was adsorbed at the end of the first pulse in scenarios I and IV. The adsorbed mass translated to 0.17–0.18 mg g^–1^ of PEG adsorption in limestone media. According to Kwarkye et al.^29^, only 4% wt. of the applied limestone material was clay, translating into 4.5–4.7 g in the limestone’s mass of 114–116 g used in this study. As these immobile clays can theoretically account for a total PEG adsorption of 1.2–1.42 mg g^–1^,^29^ the observed adsorption indicates that only 12–14% of the immobile clays were exposed, corresponding to 0.53–0.65 g of exposed clay minerals.

**Figure 2:**
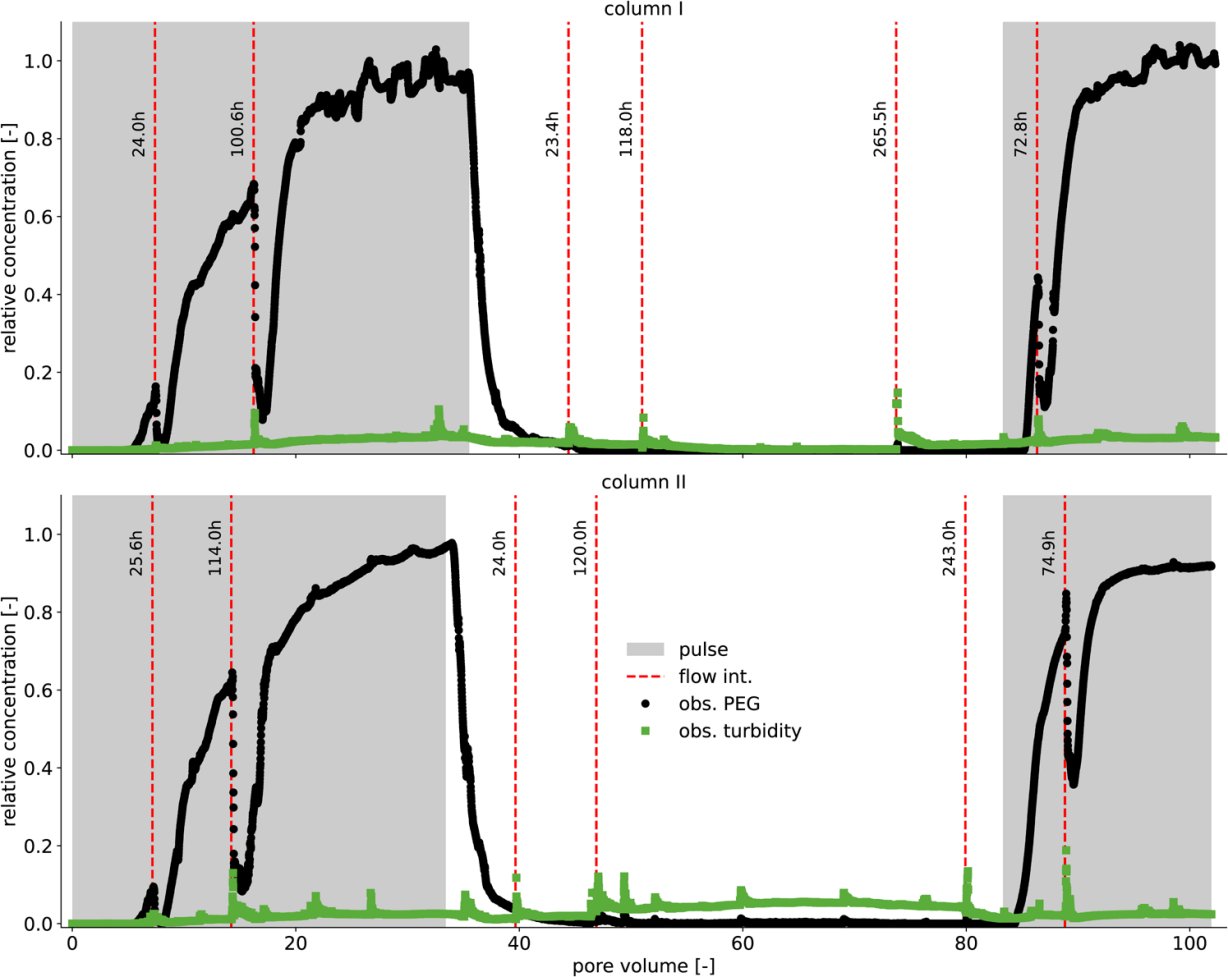
Observed PEG breakthrough with flow interruptions (black circles). Measured turbidity components during PEG transport are represented as green squares. Red-dashed vertical lines with the corresponding interruption duration in hours indicate the beginning of the flow interruption. PEG concentration was normalized to the concentration of the applied PEG stock solution, with periods of PEG pulses shaded by light gray.

**Figure 3:**
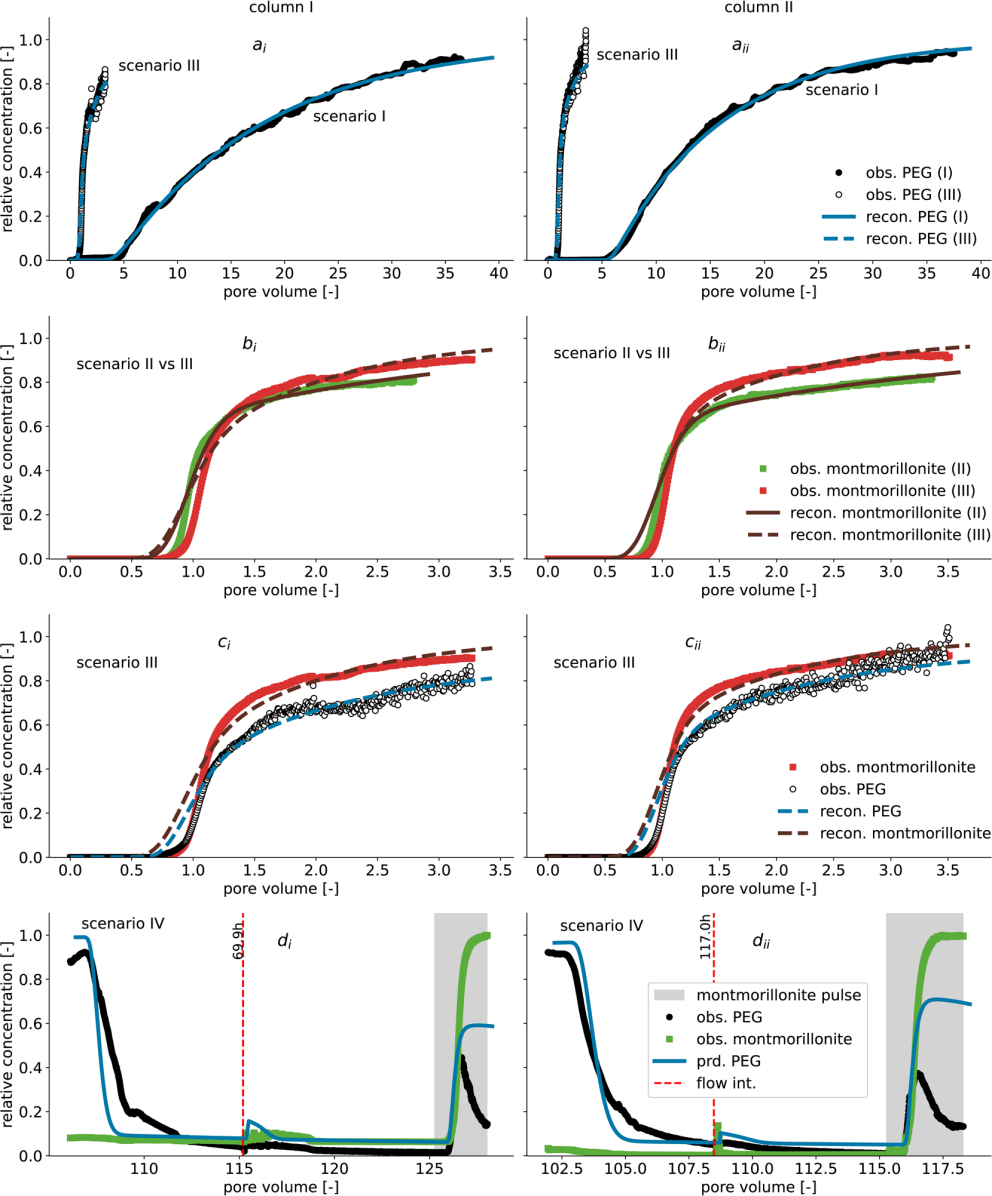
Observed PEG breakthrough compared to PEG breakthrough when transported simultaneously with montmorillonite in limestone media (a). Observed montmorillonite breakthrough compared with the breakthrough when transported simultaneously with PEG (b). Comparison of montmorillonite and PEG breakthrough following simultaneous transport in limestone media (c). Elution of PEG is displayed with black circles, and montmorillonite with green squares, and the montmorillonite pulse duration is shaded in gray (d). PEG and montmorillonite transport reconstructions are shown by solid blue and red lines, respectively. In contrast, dashed blue and dark red lines indicate facilitated transport reconstruction for PEG and montmorillonite, respectively (a, b, c). Predicted PEG release is represented by the solid blue line (d). Subscripts i and ii represent replicate columns.

Thus, PEG was strongly retarded when transported in limestone media due to adsorption on immobile clay mineral surfaces. Additionally, conducting flow interruptions in scenario IV resulted in sudden drops in effluent PEG concentration, indicating that the adsorption process was rate-limited.

### 3.5 PEG Retention in Limestone Media

A PEG elution breakthrough was conducted in scenario IV by replacing the PEG tracer solution with ultrapure water to study PEG desorption in limestone media. During the elution, PEG decreased sharply to low concentrations, pointing toward the passing of the primary elution wave shortly after (Figure 2). PEG concentration in the effluent remained negligible even after conducting flow interruptions. After the elution breakthrough, approximately 0.16–0.173 mg g^–1^ of PEG was retained in limestone media. Such strong PEG retention was also observed by Kwarkye et al.,^29^ who demonstrated that the observed PEG retention in limestone media was attributed to adsorption–desorption hysteresis. Our experiment confirmed this process by applying a second PEG tracer, resulting in a second breakthrough after the elution breakthrough (Figure 2). Unlike the first breakthrough, PEG was less retarded and appeared less dispersed. The decrease in retardation was caused by the loss of available sorption sites, which were still occupied by PEG from the previous breakthrough due to desorption hysteresis. Hence, an additional PEG mass of 5.5–6.3 mg vanished, resulting in an adsorbed amount of 0.05–0.055 mg g^–1^. Turbidity was low throughout, particularly during PEG transport. However, a slight increase in turbidity was observed immediately after flow resumed following flow interruption (Figure 2). This effect may be attributed to the rise in fluid residence times during flow interruption, which increased the content of mobile particles.

In summary, most of the adsorbed PEG did not desorb. It remained on the surfaces of immobile clay minerals, as evidenced by a decrease in the retardation of the second breakthrough (Figure 2) and the mass balance. Hence, applying PEG offers the opportunity to saturate a surface with adsorbed sorbates even if the aqueous phase returns to low concentrations of that sorbate. This can facilitate the assessment of competitive adsorption between mobile and immobile sorbents, as a well-defined surface coverage of the immobile sorbents can be achieved.

### 3.6 Transport of Montmorillonite in Limestone Media (Scenario II)

The first arrival wave of montmorillonite appeared after exchanging less than one pore volume of tracer solution (Figure 3b). This arrival was similar to that of NaCl (see Supporting Information Figure 3b), indicating a negligible retardation due to equilibrium interactions. Additionally, the mean transport velocity of montmorillonite was similar to that of NaCl, which renders montmorillonite transport more than 10x faster than PEG in limestone media (Figure 2 and Figure 3a vs Figure 3b). However, after about two pore volumes were exchanged, the breakthrough curve for montmorillonite showed a pronounced tailing. It failed to reach the maximum applied tracer concentration (Figure 3b), indicating that the interactions between montmorillonite and limestone media were strongly rate-limited. The pH of the applied solution was approximately neutral. At this pH, neutral surface charge conditions prevailed at calcite and dolomite sites of limestone, while surfaces of exposed clay minerals and montmorillonite exhibited a net negative charge.^47^ Additionally, the absence of electrolytes in the applied montmorillonite stock solution may intensify repulsive forces and induce blocking effects.^48^ Repulsive forces between montmorillonite and exposed clay minerals may reduce interactions, promoting faster transport. Besides high mobility, the presence of an energy barrier due to repulsive forces decreases the collision efficiency of colloids, such that only a few collisions result in attachment to the surfaces of the porous medium.^16^ The reduction in collision efficiency decreases the rate of colloid deposition, resulting in a tailing of the colloid breakthrough curve. In addition, the average size of montmorillonite measured with DLS decreased by about 10 nm after transport through the limestone medium. This was indicative of straining of montmorillonite particles during transport that can contribute to the observed tailing in the colloid breakthrough curve.^49^ Consequently, the observed tailing in montmorillonite breakthrough curves can be attributed to blocking effects induced by low electrolyte concentrations, reduced montmorillonite deposition kinetics resulting from repulsive forces exerted by exposed clay mineral surfaces, and straining of transported montmorillonite minerals.

In summary, montmorillonite was much more mobile in limestone media than PEG. Thus, combined with PEG’s strong affinity for montmorillonite, as shown in batch experiments, montmorillonite presents a potential carrier for PEG transport in limestone media.

### 3.7 PEG Facilitated Transport in Limestone Media (Scenario III)

Compared to scenario I, PEG was transported more than 10x faster in scenario III when introduced into the columns with montmorillonite (Figure 3a). Additionally, the PEG breakthrough appeared less dispersed and resembled that of montmorillonite, suggesting PEG was almost entirely cotransported by montmorillonite (Figure 3c). However, the observed montmorillonite breakthrough was similar to that in scenario II (Figure 3b), indicating that PEG adsorption on montmorillonite did not significantly alter its transport behavior in limestone media. Due to the nonionic character of PEG, the surface electrostatic properties of montmorillonite were not affected by PEG adsorption, resulting in a net negative charge even after PEG adsorption. Consequently, the montmorillonite-PEG association still experienced repulsive forces from exposed clay minerals in the limestone media, which PEG did not bridge, despite its considerable affinity for both montmorillonite and the exposed clay minerals at the limestone media surfaces. In effect, PEG transport was entirely controlled by the behavior of montmorillonite, as indicated by the similarity between the PEG and montmorillonite breakthrough curves (Figure 3c).

### 3.8 Release of Retained PEG by Montmorillonite (Scenario IV)

The potential mobilization of retained PEG by montmorillonite in scenario IV was investigated. This process began by rinsing the columns in scenario IV to achieve a second elution breakthrough (Figure 3), which extends the results shown in Figure 2. As with the first elution breakthrough (Figure 2), the PEG concentration in the effluent decreased sharply to low levels (Figure 3d). Additionally, flow interruptions did not cause a significant increase in PEG concentration in the effluent (Figure 3d), consistent with the observation during the first elution breakthrough in Figure 2. Hence, the desorption hysteresis, which inhibited PEG desorption in the first elution breakthrough, also persisted in the second elution breakthrough. However, there was a sudden increase in PEG concentration at the effluent during the breakthrough of montmorillonite (Figure 3), which perfectly coincided with the appearance of montmorillonite at the effluent. This suggested that, although PEG was absent in the effluent, a considerable fraction of PEG retained at the surfaces was released and cotransported by montmorillonite. It has been demonstrated that a single PEG molecule can adsorb to multiple clay mineral surfaces, thereby acting as a bridge. This formed bridge may be broken by repulsive forces when the PEG chain is short. For instance, Moudgil et al.^43^ demonstrated that long PEG chains with molecular weights greater than 1 million g mol^–1^ act as bridges between dispersed clay minerals in solution, allowing flocculation into larger aggregates. However, the molecular weight of the applied PEG in our experiments was about 4800 g mol^–1,^ ^29^ indicating a short chain length of about 74 ethylene glycol monomers. Such a chain length, therefore, renders PEG incapable of bridging montmorillonite minerals with each other or immobile clay minerals.

A deciding factor for PEG preferring adsorption to montmorillonite could be the quantity of available montmorillonite and immobile clay mineral adsorption sites in the porous medium. Yet, in scenarios I and IV, about 0.53–0.65 g of immobile clay minerals were exposed, which was more than an order of magnitude greater than the mass of montmorillonite contained in the total water volume inside the column (0.026–0.029 g). However, the estimated adsorption capacity of montmorillonite was 3 orders of magnitude higher than that of limestone in batch experiments. Therefore, montmorillonite’s adsorption capacity was 2 orders of magnitude higher than that of the porous medium.

In summary, scenarios III and IV indicated mobilization of PEG when preadsorbed to montmorillonite or when montmorillonite is introduced into porous media after PEG adsorption. These scenarios demonstrated carrier-facilitated transport by in situ-released, mobile sorbents of allochthonous origin. In all cases, the observed PEG transport differed significantly from that observed in scenario I and in the part of scenario IV not affected by a carrier (Figure 2 and Figure 3a). Hence, it is crucial to consistently account for carrier-facilitated transport in transport models, including competitive adsorption between mobile and immobile sorbents.

### 3.9 Reconstruction of Transport Behavior

A proper representation of carrier-facilitated transport in transport models is required to reconstruct and predict PEG transport. The data across all scenarios provide a wealth of insights into individual sensitivities to specific aspects of cotransport, laying the foundation for a comprehensive parametrization and validation of carrier-facilitated transport. Here, we will present an approach for sequential parametrization that is both robust and reproducible. In all cases, dispersion coefficients were determined inversely from NaCl breakthroughs in the respective scenarios (see Supporting Information Table 1). Reconstruction of PEG transport in scenario I also required specification of sorption parameters for equilibrium and nonequilibrium adsorption. An adsorption capacity of 0.2 mg g^–1^, following Kwarkye et al.,^29^ was assumed. At the same time, isotherm constants (*K* ^*eq*^ and *K* ^*neq*^) and mass exchange coefficient (α_*f*_) were fitted, as Vereecken^36^ showed that batch-derived isotherm constants differ from what is estimated under transport conditions. The reconstructed PEG breakthrough in scenario I closely matched the observed breakthrough (Figure 3). Rate-limited mass exchange dominated adsorption, and 85–90% of surfaces contributed to nonequilibrium adsorption (Table 1). Additionally, a slow mass transfer rate coefficient of 0.007–0.008 h^–1^ was estimated, corresponding to Damköhler (Da) numbers of 1.3–1.5. These Da numbers correspond to a transport regime in which the rate of mass exchange reactions is low in consideration of the mean tracer residence time.^24,50^ Such a slow mass transfer rate required 125–145 h to attain adsorption equilibrium, resulting in the dispersed shape of the predicted breakthrough (Figure 3a).

The transport of montmorillonite in scenario II was reconstructed with high accuracy (Figure 3b) by fitting only the deposition frequency (Γ) and the deposition rate (*k*). A deposition rate of 0.03 ± 0.002 h^–1^ was estimated (Table 1), corresponding to Da numbers of 0.8–0.83, indicating that the time corresponding to the deposition rate was approximately 20% smaller than the mean montmorillonite residence time in limestone media. This indicated most collisions of montmorillonite during transport did not cause a deposition effectively decreasing the collision efficiency.^16^

The parameters of Scenarios I and II were applied to reconstruct PEG and montmorillonite transport in Scenario III. This also required fitting the isotherm constant (*K* ^*mt*^) of PEG to montmorillonite, as the adsorption capacity obtained from batch experiments was used as previously. Additionally, PEG-montmorillonite association deposition parameters (*K*_*b*_^*neq*^ and α_*b*_) were fitted using observed montmorillonite breakthroughs in scenario III, which allowed a consideration of potential modifications of montmorillonite deposition characteristics after PEG adsorption. PEG and montmorillonite transport were reconstructed with striking agreement with observed transport in scenario III (Figure 3c). Additionally, transport parameters were estimated with small uncertainty, indicating that the model accurately described the critical transport processes accounting for carrier-facilitated transport. From the estimated adsorption capacity for PEG adsorption to montmorillonite in batch experiments, about 8.8 mg of PEG was required to saturate all the adsorption sites of montmorillonite contained in the column aqueous phase. At the maximum applied PEG concentration, the column aqueous phase contained approximately 1.5 mg of PEG, about 6x less than the amount required to achieve total adsorption on montmorillonite. In effect, most PEG was adsorbed onto montmorillonite when applied to the columns. However, from the estimated mass balance in scenario I, the limestone medium contained more immobile adsorption sites than the quantity of montmorillonite in the aqueous phase. Yet, the Langmuir isotherm constant estimated for PEG adsorption to montmorillonite (Table 1) ranged from 4 to 15 L mg^–1^, indicating a strong adsorption affinity. This adsorption affinity was almost twice as high as that to immobile clays in column 1 and nearly 3 times higher than that of column 2. Thus, in addition to its higher adsorption capacity, PEG’s strong sorption affinity for montmorillonite may have contributed to its sustained adsorption during transport through limestone media.

In summary, the fitted parameters had low uncertainty and narrow confidence intervals, indicating the robustness of the sequential model parametrization (Table 1). Transport parameters such as mass exchange rate, isotherm constant, and adsorption capacity may be crucial for competitive adsorption between mobile and immobile sorbents. However, the hierarchy of importance of these parameters for competitive adsorption is not yet clear and therefore needs further investigation through a sensitivity analysis.

### 3.10 Independent Transport Prediction

Scenario IV was accurately predicted using the model from scenario III. Without fitting any parameters, the model could predict the release of retained PEG following the introduction of montmorillonite (Figure 3d). The consideration of montmorillonite in the model led to PEG desorption from immobile surfaces. This caused instantaneous desorption of PEG mass from equilibrium adsorption sites and increased desorption from nonequilibrium adsorption sites. With a high adsorption capacity, most of the exported PEG was associated with montmorillonite, leading to a predicted PEG breakthrough that coincided with the observed montmorillonite breakthrough and matched the point of PEG release. However, the model overestimated the concentration of released PEG, which may be attributed to differences in mass transfer rates between scenarios, the lack of a proper representation of hysteresis in the model, and the exposure of new adsorption sites.

In summary, the estimated adsorption parameters for PEG adsorption to montmorillonite could also cause already adsorbed PEG to be released when montmorillonite was introduced later in the model. This indicates that the cotransport of PEG with montmorillonite is favorable, even if PEG is adsorbed onto immobile sorbents.

### 3.11 Sensitivity of Sorption Parameters

To comprehensively identify conditions that favor carrier-facilitated transport, a sensitivity analysis is required that compares the adsorption parameters of montmorillonite and immobile clay minerals over a broader range of parameters as presented in the experiments. The adsorption parameters of montmorillonite and immobile clay minerals were compared under different nonequilibrium sorption conditions. This was achieved through forward simulations, in which PEG was preadsorbed onto montmorillonite with varying adsorption affinities and montmorillonite-to-immobile-clay adsorption capacity ratios. A considerable influence of carrier-facilitated transport emerged from the models when PEG adsorption affinity to montmorillonite was equal to its affinity to immobile sorbents (Figure 4, K* = 1). However, PEG was retained in the column at K* < 1 even after increasing montmorillonite’s adsorption capacity to 3 orders of magnitude higher than that of the immobile sorbent (Figure 4 and Supporting Information Figures 5 and 7). At K* ≥ 1, an increase in montmorillonite adsorption capacity increased the cotransported PEG. This suggests that while the amount of adsorption sites is essential to transport more adsorbed sorbate, the adsorption affinity to mobile sorbents must be significantly higher than the affinity to immobile sorbents to allow carrier-facilitated transport to occur. At low aqueous concentrations where the shape of Langmuir sorption isotherms is approximately linear, carrier-facilitated transport only depends on the affinity of the adsorbate to the carrier. Without the limitation of the maximum adsorption capacity, sorption to mobile sorbents following a linear or a Freundlich isotherm can result in the transport of a considerable amount of adsorbate when sorption affinity to mobile sorbents is greater than that of immobile sorbents. This observation is consistent with the study by Xing et al.,^51^ which found increased colloid-facilitated transport of pharmaceutical and personal care products with higher adsorption affinity to mobile sorbents. On the other hand, higher affinity to immobile sorbents can retain adsorbate and reduce carrier-facilitated transport. However, a considerable amount of cotransported PEG was predicted at K* < 1 when Da was less than unity (see Supporting Information Figure 5). The mass transfer rate at Da lower than unity is slow, such that even high adsorption capacity and affinity of PEG for the immobile sorbent were insufficient to retain PEG in the column. In turn, weak adsorption affinity to mobile sorbents could still contribute to carrier-facilitated transport. In particular, sorption behavior following a linear or a Freundlich isotherm with infinite adsorption sites can result in considerable carrier-facilitated transport at high transport velocities. For instance, Schäfer et al.^52^ observed increased mobilization of radionuclides by bentonite with increasing flow velocity. This is crucial in natural systems since Da decreases with increasing flow velocity. During ponding infiltration or inundation conditions, high flow velocity along preferential flow paths^1,53^ can intensify carrier-facilitated transport.

**Figure 4:**
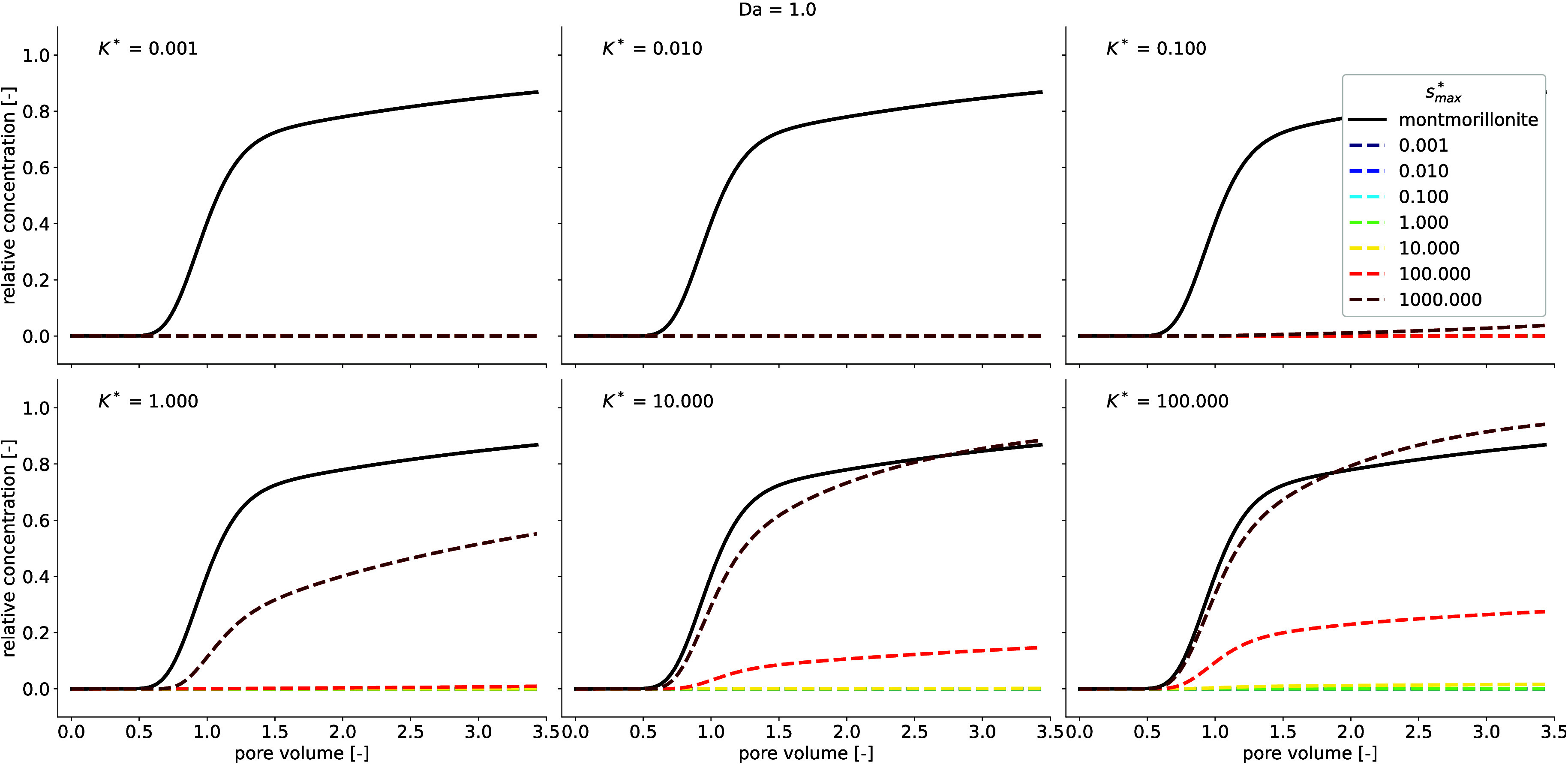
Forward simulations comparing Langmuir isotherm constants and adsorption capacity, defining PEG adsorption to montmorillonite and immobile sorbents. The solid black line represents the breakthrough of montmorillonite, while the dashed lines represent that of PEG. Dashed lines are color-coded from blue to red, covering the minimum to the maximum value of *s*_max_^*^ (the ratio of montmorillonite adsorption capacity to the adsorption capacity of limestone). The simulation was performed at Da = 1.

## 4 Environmental Relevance

Carrier-facilitated transport often mobilizes pollutants, such as PAHs, pesticides, and heavy metals, that may otherwise be adsorbed to the soil matrix.^8,10,50^ Additionally, carrier-facilitated transport can be explored to facilitate a controlled release of pollutants, reducing potential mobility to water resources.^11,23^ Yet, a quantitative assessment of carrier-facilitated transport is challenging due to ambiguities in accurately parametrizing carrier-facilitated transport models. We approached this challenge by conducting a series of transport experiments that enabled the sequential determination of transport parameters with minimal uncertainty. This resulted in accurate predictions of carrier-facilitated transport behavior conducted under distinct experimental conditions. Such a combination of modeling and experiment is instructive for other model-inversion problems that suffer from parameter correlation. In a sensitivity analysis, we observed that carrier-facilitated transport may intensify under conditions of ponding infiltration. Furthermore, extreme weather events, such as intense precipitation that leads to flooding, threaten groundwater resources, as large quantities of carrier-associated pollutants can be mobilized. Additionally, pollutants with high affinity to mobile sorbents can be easily mobilized and transported to the deeper subsurface. These factors underscore the need to account for carrier-facilitated transport in remediation. In the current study, we present experimental and modeling approaches to allow an accurate consideration of carrier-facilitated transport in such measures. Besides this, the observed influence of sorption affinity to mobile and immobile sorbents, as well as enhanced carrier-facilitated transport in response to sorption kinetics at immobile sorbent surfaces, aligns with existing literature.^7,51,52,54^ Thus, similar carrier-facilitated transport behavior may apply even when the sorption process follows different isotherms like the linear or the Freundlich isotherm. The current study demonstrates that an accurate representation of sorbate adsorption to mobile and immobile sorbents is the basis for assessing carrier-facilitated transport and competitive sorption between mobile and immobile sorbents. This assessment was possible because PEG’s affinity for clay minerals can be translated to the sorption of other compounds with high adsorption affinity such as PAHs, heavy metals, pharmaceuticals, and pesticides. Therefore, the application of PEG allowed a quantitative assessment and a general understanding of factors controlling carrier-facilitated transport in natural porous media. The study employed limestone materials but could also be extended to other porous media with reactive silicate surfaces, such as quartz sand. For instance, Ritschel et al.^26^ observed strong PEG adsorption on pure quartz sands and proposed the application of PEG to quantify reactive silicate surfaces. Additionally, carrier-facilitated transport and competitive adsorption with other mobile and immobile sorbents, such as iron oxides, may be explored by modifying the PEG end-group functionality.^42^ We observed no significant changes in the transport behavior of montmorillonite after adsorbing PEG. However, experiments with different ratios of PEG concentration to montmorillonite, as well as PEG with varying chain lengths, may help identify how mobile sorbates influence the transport of mobile sorbents. Finally, the model may be extended to account for other sorption processes, such as adsorption–desorption hysteresis and the exposure of new adsorption sites,^29^ which may also increase carrier-facilitated transport.

## Supplementary Material



## Notes

Additional experimental details, methods, and results including protocol for extracting montmorillonite from bentonite, SEM image and FT-IR spectra of montmorillonite, description and overview of activities conducted during column experiments, figures showing measured NaCl breakthroughs, and extended results of sensitivity analysis performed at different Damköhler numbers (PDF)
