# Supplementary material for: Competitive Sorption and Carrier-Facilitated Transport of Organic Polymers by Clay Minerals in Limestone Media: Experimental Evidence and Numerical Analysis
Source: Environ Sci Technol. 2026 Jan 28;60(5):4309–20. doi: 10.1021/acs.est.5c09934 (PMC12895513; doi:10.1021/acs.est.5c09934)
Supplement: Supplementary file 1 [file es5c09934_si_001.pdf]

Supporting Information

Competitive sorption and carrier-facilitated transport of organic polymers by clay minerals in limestone media: experimental evidence and numerical analysis

Nimo Kwarkye[1], Thomas Ritschel[1], Andreas Pihan[1], Kai U. Totsche[1,2,*]

[1]Department of Hydrogeology, Friedrich-Schiller-University Jena, Burgweg 11, 07749 Jena, Germany

[2]Cluster of Excellence Balance of the Microverse, Friedrich-Schiller-University Jena, Fürstengraben 1, 07743 Jena, Germany

Correspondence (*): kai.totsche@uni-jena.de

Methods

Extraction of montmorillonite from bentonite

Montmorillonite extracted from bentonite was used as a mobile sorbent in column experiments and as a substrate in batch experiments. The bentonite, which was in rock form, was crushed and homogenized into clasts <200 µm. The homogenized bentonite was suspended in deionized ultrapure water using an ultrasonic probe (Sonifer W-250 D, Branson Ultraschall GmbH, Füth, Germany) at 60% amplitude for approximately 16 minutes. The clay fraction < 2 µm was separated by applying the Atterberg sedimentation approach. The required time for the sedimentation of particles with a size greater than 2 µm was estimated using the Stokes equation. The supernatant, mostly consisting of montmorillonite with a size < 2 µm, was extracted and freeze-dried. Once extracted, montmorillonite was treated with sodium chloride (NaCl, Roth, Germany) to exchange calcium ions with sodium ions, resulting in sodium montmorillonite. The average size of montmorillonite was determined using dynamic light scattering (DLS) (Nano ZS, Malvern Panalytical Ltd, Malvern, UK). Additionally, SEM measurements were conducted to characterize the physical features of montmorillonite, including its shape and structure.

Column experiment setup

Table 1. Estimated properties of columns used in transport experiments in scenarios I, II, III, and IV. Dispersion values were obtained by fitting sodium chloride breakthroughs conducted before every scenario experiment.

	Bulk density (mg L^{-1})	Dispersion (cm^2 h^{-1})	Water content	Flow velocity (cm h^{-1})	Column length (cm)
Scenario I (column 1)	1.68	0.36 ± 0.02	0.36	0.28	7.9
Scenario I (column 2)	1.62	0.29 ± 0.03	0.38	0.26	7.9
Scenario II (column 1)	1.67	0.12 ± 0.02	0.37	0.16	7.4
Scenario II (column 2)	1.66	0.14 ± 0.035	0.37	0.195	7.4
Scenario III (column 1)	1.67	0.14 ± 0.03	0.36	0.186	7.4

Scenario III (column 2)	1.66	0.14 ± 0.04	0.37	0.2	7.4
Scenario IV (column 1)	1.61	0.18 ± 0.04	0.39	0.4	7.7
Scenario IV (column 2)	1.64	0.14 ± 0.04	0.38	0.4	7.7

Table 2. Experimental details of observation data applied in scenario IV. FI represents flow interruption, which is defined by the time of interruption in hours and pore volumes, as well as the duration of the interruption. The beginning of forward and elution breakthroughs is indicated by Pulse and Elution, respectively.

Event	Time (h)	Pore vol (-)	duration (h)
column I			
PEG Pulse	0.0	0.0	-
FI	55.3	7.4	24
FI	145.2	16.2	100.6
Elution	389.9	35.5	-
FI	456.3	44.4	23.4
FI	528.8	51.0	118.1
FI	816.7	73.7	265.5
PEG Pulse	1153.6	83.3	-
FI	1176.4	86.3	72.8
FI	1368.4	102.3	2137.2
Elution	3534.1	106.1	-
FI	3602.0	115.2	69.9
Mt Pulse	3746.9	125.2	-
column II			
PEG Pulse	0.0	0.0	-
FI	52.5	7.2	25.6
FI	129.7	14.2	114.0
Elution	384.2	33.4	-
FI	429.9	39.7	24.0
FI	506.9	46.9	120.0
FI	868.7	79.9	243.0
PEG Pulse	1136.4	83.3	-
FI	1177.0	88.8	74.9
Elution	1348.0	101.9	-
FI	1395.8	108.5	117.1
Mt Pulse	1562.6	115.3	-

Results

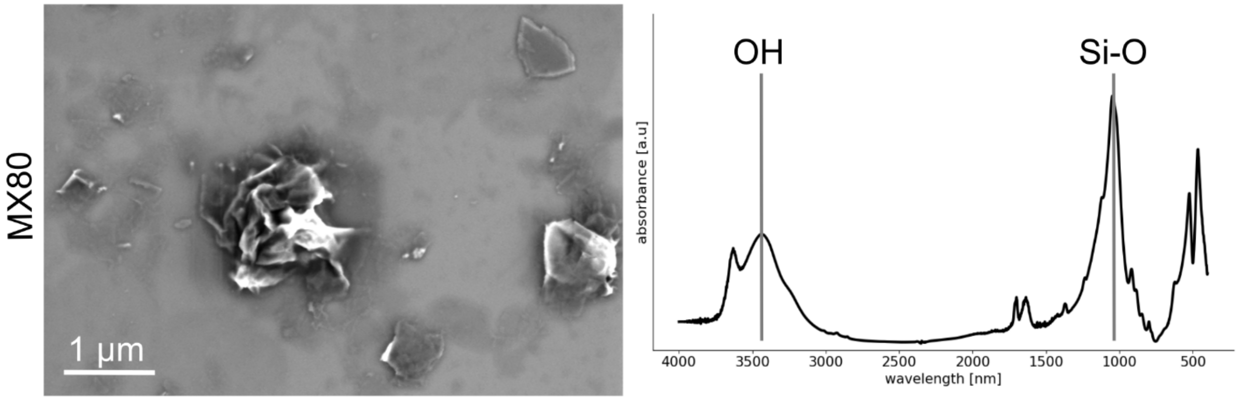

Figure 1. SEM image of montmorillonite showing plate-like features (left) and FT-IR spectra of montmorillonite (right). Vertical lines indicate Si-O and OH stretching.

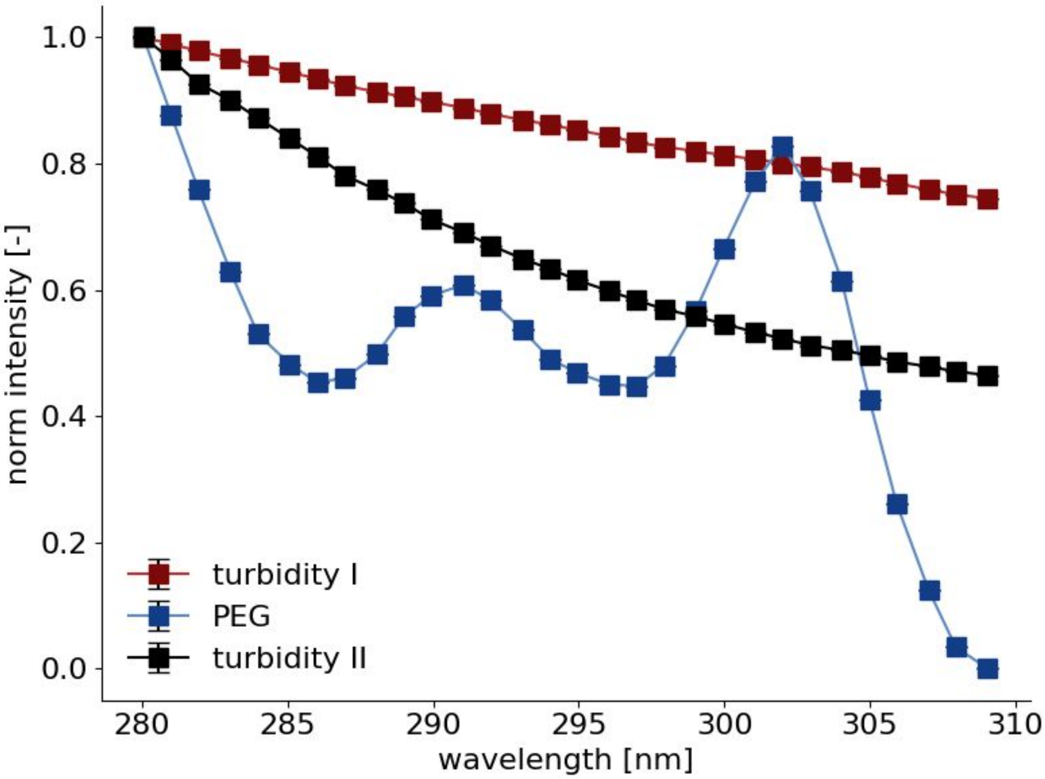

Figure 2. PEG and turbidity components used in a linear combination fit to estimate montmorillonite and PEG concentration in the aqueous phase.

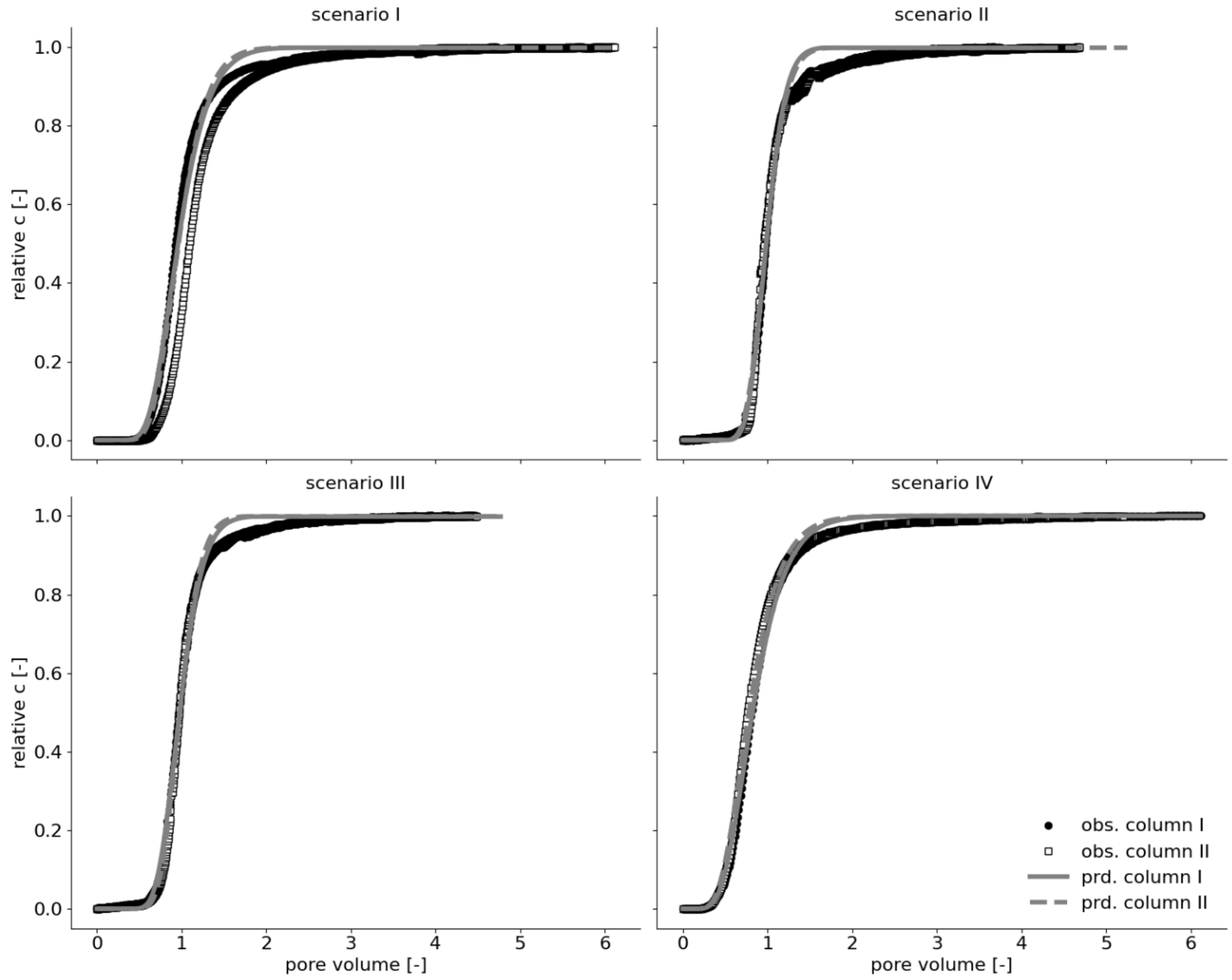

Figure 3. The results of the sodium chloride breakthrough conducted before each scenario experiment, along with the fitted model used to estimate dispersion, are presented.

Table 3. Results of fitted mass transfer rate coefficient while fixing all other transport parameters in scenario IV.

scenario IV	α (h-1)
column I	0.01 ± 0.0002
column II	0.014 ± 0.000005

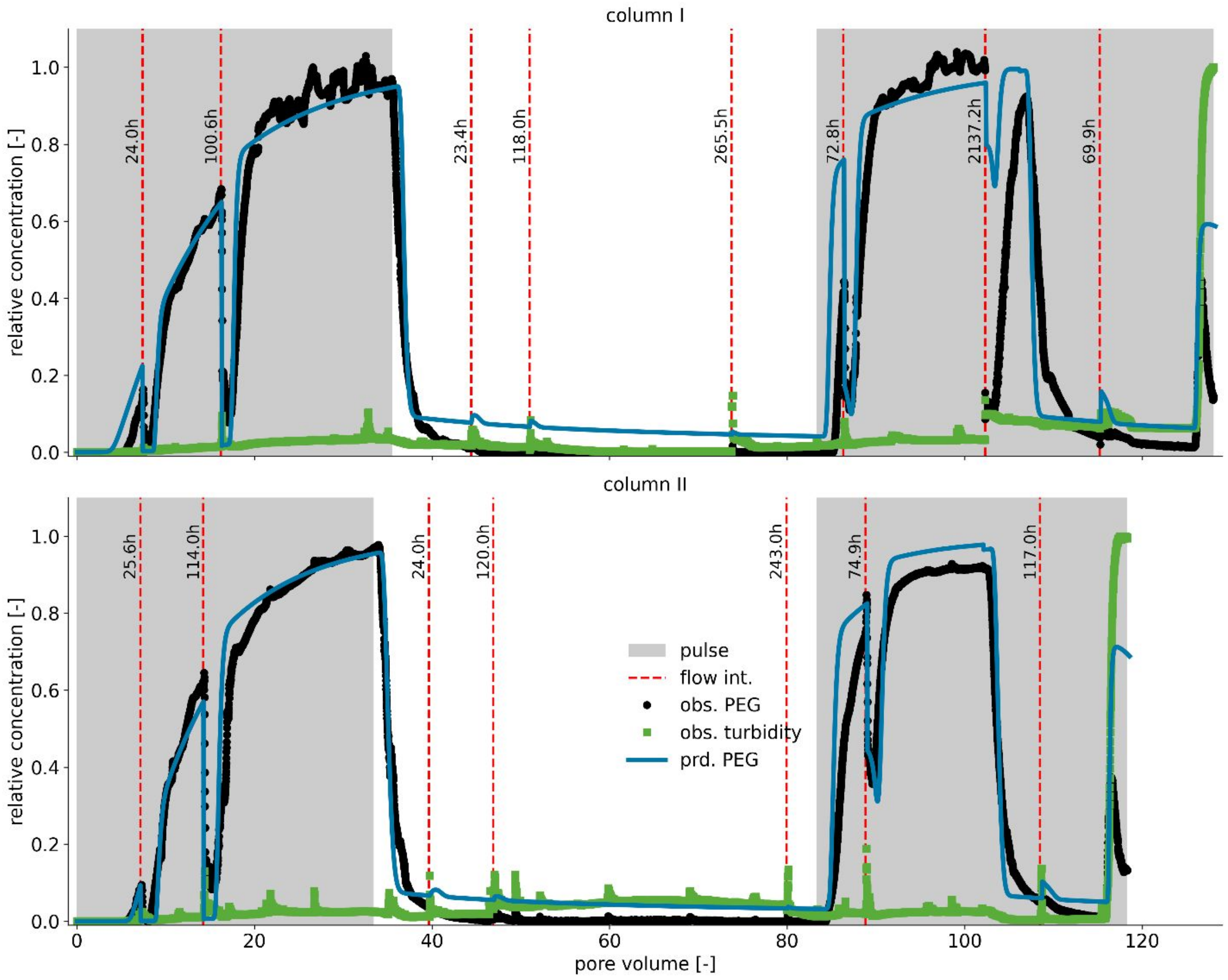

Figure 4. The results of PEG transport prediction in scenario IV after fitting mass transfer rate coefficients accounting for non-equilibrium PEG adsorption to immobile surfaces.

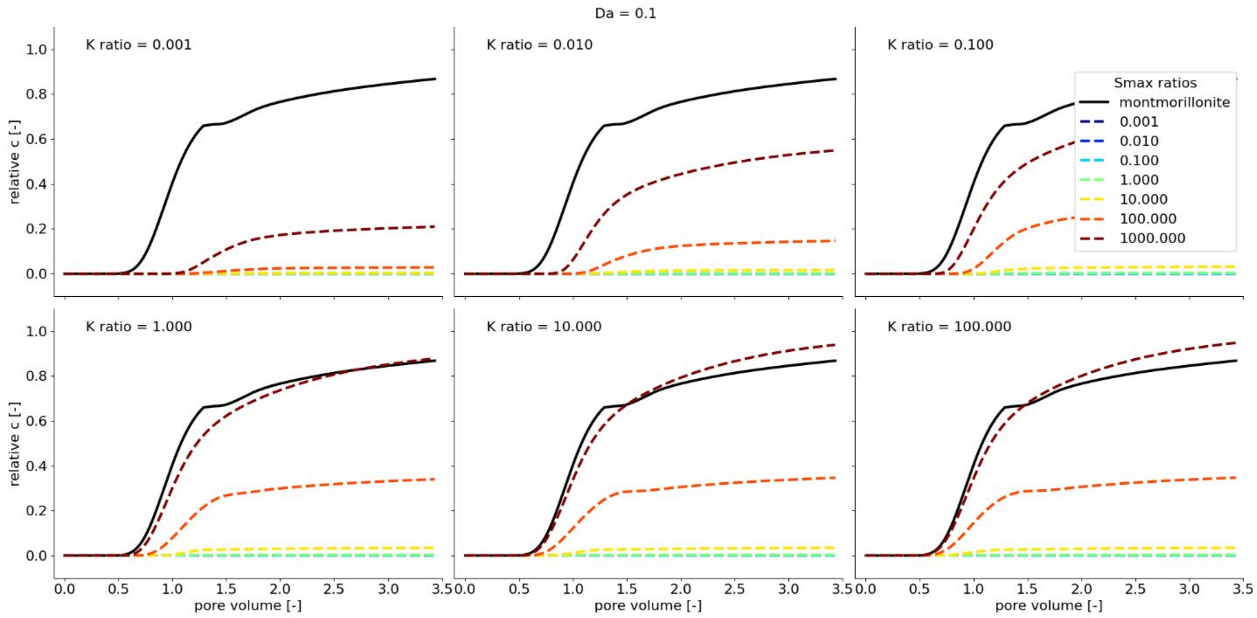

Figure 5. Forward simulations comparing Langmuir isotherm constants and adsorption capacity defining PEG adsorption to montmorillonite and immobile sorbents. The solid black line represents the breakthrough of montmorillonite, while the dashed lines represent that of PEG. Dashed lines are color coded from blue to red, covering the minimum to the maximum value of s^*_{max} (the ratio of montmorillonite adsorption capacity to the adsorption capacity of limestone). The simulation was performed at Da = 0.1.

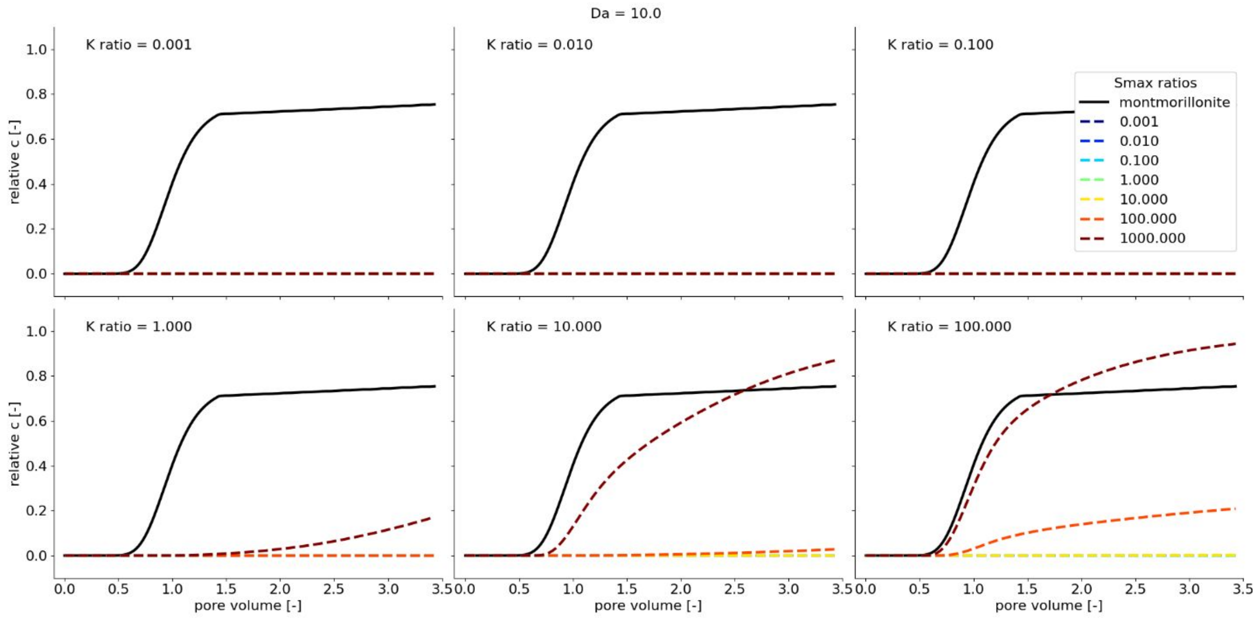

Figure 6. Forward simulations comparing Langmuir isotherm constants and adsorption capacity defining PEG adsorption to montmorillonite and immobile sorbents. The solid black line represents the breakthrough of montmorillonite, while the dashed lines represent that of PEG. Dashed lines are color coded from blue to red, covering the minimum to the maximum value of s^{*}_{max} (the ratio of montmorillonite adsorption capacity to the adsorption capacity of limestone). The simulation was performed at Da = 10.

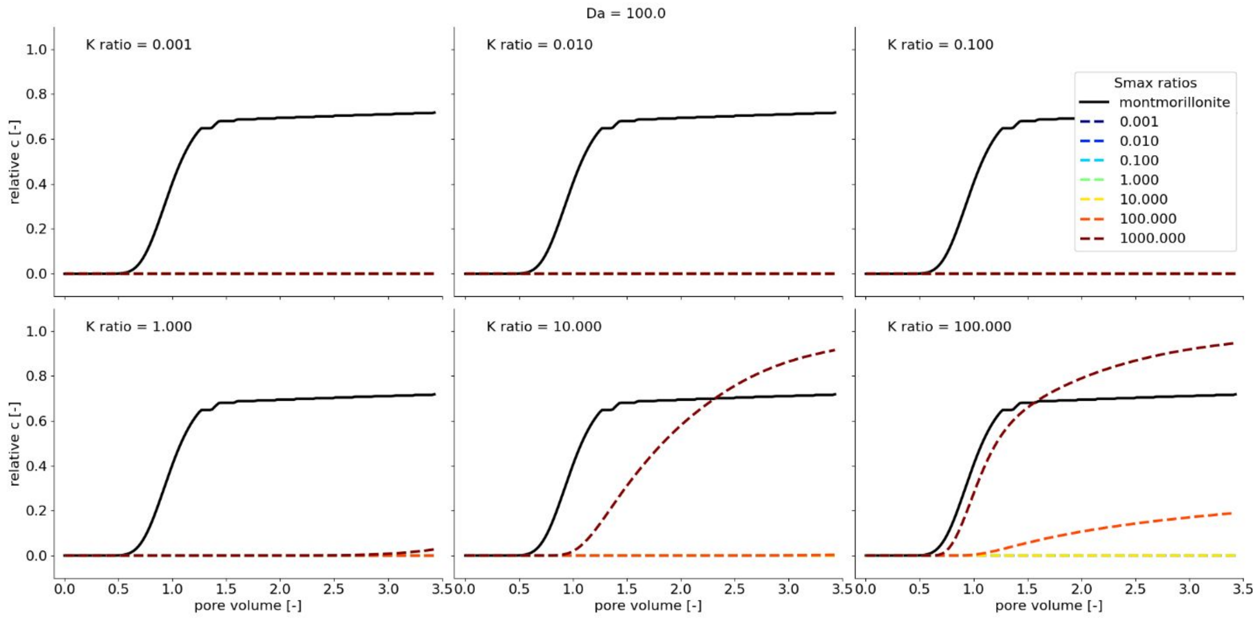

Figure 7. Forward simulations comparing Langmuir isotherm constants and adsorption capacity defining PEG adsorption to montmorillonite and immobile sorbents. The solid black line represents the breakthrough of montmorillonite, while the dashed lines represent that of PEG. Dashed lines are color coded from blue to red, covering the minimum to the maximum value of s^*_{max} (the ratio of montmorillonite adsorption capacity to the adsorption capacity of limestone). The simulation was performed at Da = 100.
